# Impact of COVID-19 Pandemic on Total Mortality in Poland

**DOI:** 10.3390/ijerph18084388

**Published:** 2021-04-20

**Authors:** Kamil Barański, Grzegorz Brożek, Małgorzata Kowalska, Angelina Kaleta-Pilarska, Jan Eugeniusz Zejda

**Affiliations:** Department of Epidemiology, Poland Faculty of Medical Sciences in Katowice, Medical University of Silesia in Katowice, 40-752 Katowice, Poland; gbrozek@sum.edu.pl (G.B.); mkowalska@sum.edu.pl (M.K.); akaleta@sum.edu.pl (A.K.-P.); jzejda@sum.edu.pl (J.E.Z.)

**Keywords:** COVID-19, mortality, estimation, coronavirus, Poland, Silesia

## Abstract

Background: According to published data the number of deaths attributed to COVID-19 is underestimated between 30 and 80%. Aim: The aim of this study is to assess the impact of COVID-19 on total mortality of Poland and the Silesian voivodship. Methods: Secondary epidemiological data on COVID-19 deaths were obtained from the Ministry of Health registry and data on total mortality were gathered from the National Statistical Office and Registry Office in Poland. Three scenarios were used to estimated COVID-19 deaths: real number + an extra 30%, 60%, and 70% excess total deaths. Results: In 2020, there were 73,254, 64,584, and 67,677 excess deaths in comparison to 2017–2019, respectively. For the Silesian voivodship, it was 8339, 7946, and 8701, respectively. The total mean increase in deaths was 16% for the whole country and the Silesian voivodship. The simulation for 30% extra COVID-19 deaths gave COVID-19 mortality equal to 12.5%; *n* = 50,708 deaths, for extra 60%; 17.9% *n* = 72,866 and for extra 70%; 19.7% *n* = 80,251 for Poland; and 11.9% (*n* = 6072), 17.2% (*n* = 8740), 24.2% (*n* = 12,297), respectively, for the Silesian voivodship. Conclusions: The participation of COVID-19 in total deaths should not exceed 20% for Poland and 24% for the Silesian voivodship in 2020.

## 1. Introduction

In Poland (pop. 37,672,367), the first confirmed case, as well as the first death of COVID-19, was reported in March 2020. Initially, the spread of infection was slow with the highest numbers of infections recorded in the Silesia voivodship (pop. 4,517,635, density 3663 people/km^2^, a highly industrial region on South of Poland) [1].

The registered number of deaths in Poland attributed to COVID-19 in 2020 was 28,551. At the same time, data regarding new cases of infections of COVID-19 showed the number to be 1,267,051 (31 December 2020). The COVID-19 fatality case ratio was 2.2%. Currently (26 February 2021), case fatality ratio is 2.5%. In 2020, during the COVID-19 pandemic, the total number of deaths in Poland increased to 71,541 in comparison to 2019. Globally, in Poland in 2020, 1.26% people died; this was an increase of 0.18% compared to 2019 [2]. In Poland, the number of deaths as well as mortality rates in 2017, 2018, and 2019 were stable and did not differ significantly. There is no doubt that the pandemic increased the general and specific deaths globally, as well as observed in Poland and the Silesian voivodship [3]. Current published data regarding COVID-19 suggest an underestimation of the number of deaths attributed to COVID-19 in different World locations by at least 35% [4]. Table 1 presents some estimates of COVID-19 attributed deaths.

In addition, our population base study showed that the real number of COVID-19 infection in Silesia could be even four times higher than official statistics [9].

The lack of estimates regarding the potential underestimation of COVID-19 mortality in Poland, as well as the fact that the Silesian voivodship noticed one of the highest numbers of COVID-19 cases in Poland, were the reasons why we performed real number of COVID-19 death estimation based on analysis of available data.

## 2. Materials and Methods

The national reports regarding the number of COVID-19 deaths are available and updated daily on the Polish Ministry of Health’s website. The reports contain information about the number of deaths by months, age, sex, and regional location (voivodships). Data about total mortality regardless of the cause of death are available from National Statistical Office reports and the national registry office. Data describing the monthly number of total deaths from 2017 to 2020 for Poland and the Silesian voivodship were used in the analysis. According to published results [4,5,7,10] three scenarios for simulating the impact of COVID-19 on global mortality were used. The first scenario adds an extra 30% to the number of reported COVID-19 deaths (simulated COVID-19) from excess deaths, the second scenario adds an extra 60% (simulated COVID-19) from excess deaths to all reported COVID-19 deaths, and the third adds an extra 70% in total and by months (according to Ministry of Health reports). Our analysis and simulations covers the data collected between 4 March 2020 (first recorded COVID-19 case) and 31 December 2020 year. The reference point for estimating changes in year 2020 was a mean number of death from 3 years (2017, 2018, and 2019).

## 3. Results

### 3.1. Number of Deaths in Poland and Silesian Voivodship

In Poland, the total number of deaths (all causes of death) in 2017 was *n* = 405,404; in 2018 *n* = 414,074; in 2019 *n* = 410,981; and in 2020 *n* = 478,658. The average number of deaths in 2017–2019 was *n* = 410,153 and increased by 16.7% in 2020 (Table 2). The number of total deaths in the Silesian voivodship was stable between 2017 and 2019 (this number did not cross 53,000 in each of those years). In 2020, the yearly total mortality rapidly increased by 16%, in comparison to the average number of deaths in a previous period (Table 3).

### 3.2. Simulated Number of Deaths

From 2017 to 2019, the average number of death was 410,153, while in 2020 the number of all deaths was 478,658. This shows a total of 68,505 excess deaths. In 2020, there was a 16% increase in total mortality. Data representing the population of the entire voivodship and the country as a whole are useful for the estimation of undocumented mortality due to COVID-19. As a direct measure of the real number of COVID-19 death, it was not possible, indirect methods were applied to estimate the size of this problem. We based on the estimation methods used by other authors [10,11,12,13,14,15]. From 8 March 2020 to 31 December 2020 the number of deaths in Poland was 407,120 including 73,858 deaths reported as the excess number of death. In 2020, there were 28,551 deaths from COVID-19. At the same time, the frequency of COVID-19 deaths was 7% from all deaths in 2020. Since the current literature suggest an underestimation of deaths attributed to COVID-19 death at least 35% [4] we proposed three scenarios where we add extra 30%, 60%, and 70% deaths from COVID-19 to officially reported deaths from COVID-19.

The results of our simulation showed that COVID-19 was a cause of 12.5% all deaths in 2020 (while considering 30% extra deaths from excess deaths). In case of a simulation considering 60% of additional deaths, COVID-19 caused 17.9% of all deaths in 2020. In the third scenario, we used the same quantification for the last simulation as reported by the Polish Ministry of Health, which was 70% [11] and COVID-19 was a reason of 19.7% of all deaths in 2020 was 19.7% (Table 4). In a parallel simulation conducted for the Silesian voivodeship, while considering 30%, 60%, and 70% extra deaths from excess death, COVID-19 was a cause of 11.9%, 17.2%, and 24.4% all deaths in 2020 respectively Table 5.

## 4. Discussion

The increased number of total death in 2020 is noticed in different World locations and is an undisputable phenomenon [16]. The only question is, what is the scale of additional death increase, and how many of that excess deaths are attributed to COVID-19 infections. In Poland, in comparison to the average number of deaths from 2017 to 2019, it was 17% increase, while in the Silesia voivodship it was 16%. Month-to-month (2020 vs. mean 2017–2019) comparison of deaths showed the different scale of excess deaths from 1 to 91% with an increased proportion in the second half of 2020. Such a situation was characteristic for Poland as well as for the Silesian voivodship.

In Belgium, the total increase in excess deaths attributed to COVID-19 accounted for 96% [17]. In the USA, the number of excess all-cause deaths was 28% higher than the officially reported COVID-19 deaths during that period [10], others have suggested a lower number of excessed deaths [12].

Several countries had more than double the historical weekly all-cause mortality during the peak of the pandemic, including Ecuador (497%), Peru (304%), Spain (257%), and the UK (201%), whereas some countries did not across 200%, e.g., Chile (170%), France (159%), Italy (185%), the Netherlands (175%), South Africa (165%), and Sweden (150%). Denmark, New Zealand, and Norway had fewer deaths in 2020 compared to the previous years [4]. The detailed results are included in Table 1 from Kung’s study which was the basis of the methodology of our paper.

The conclusion in the article provided by Kung et al. was that the “number of deaths attributed to COVID-19 are underestimated by at least 35%” [4]. In our study, we suggested that COVID-19 could cause no more than 19.7% of all deaths in Poland in 2020. We performed a simulation for Poland, with emphasis on the Silesian voivodship, a densely populated region in Southern Poland. Covid-19 caused an estimated 24% of all total number of deaths in the Silesian voivodship, which corresponds with results of time-delay estimation that accounted for 20% performed elsewhere [13]. That could be related to the highest incidence rate of COVID-19 in a chosen region, and at the same time, the region with the highest number of deaths because of COVID-19.

On the other hand, it is worth noting that the COVID epidemic caused, in practice, total inefficiency of the health care system in Poland, with very limited access not only to specialist, planned diagnostic procedures or operations but also to basic family doctors. We expect that consequences of this situation must increase the number of deaths not related directly to the COVID-19 infection.

The limitation of our study is lack of access to the death certificates. These are simulations base on officially published data, but at this moment, there are no better source of the data in Poland. Unfortunately, currently available data do not allow us yet to perform plausible separate simulations for Poland according to gender or different age groups. Our research based on data from hospital deaths in Silesia revealed that the risk of death in COVID-19 hospitalized patients is greater in men than in women (OR (Odds ratio), 1.35; 95% CI, 1.07–1.70), and increases with age and with the number of coexisting diseases (mostly with chronic cardiovascular, respiratory, and metabolic diseases) [18].

Another limitation of our simulation is that the number of excess deaths did not include COVID-19 testing before the beginning of the epidemy. However, we included in our calculation the only period in which the Polish government officially started testing against COVID-19. Another limitation of our study is that some people may have died of COVID-19 but were not diagnosed because of situation the pandemic presented [13]. In some populations, it was even 50% [15]. As we showed in our previous research, the real number of COVID-19 infections in the Silesia region (11.5%) may be four times higher as an official prevalence (2.8%) [9]. The other issue is reserved for problems with giving a proper reason for death because some direct deaths attributed to COVID-19 may be assigned to other causes of death. There might be plenty of reasons for such situation like physician mistake (due to overwork), inability to test every patient for COVID-19, and finally, time lag between infection, recovery, and cause of death.

In our simulation, we were not able to include indirect consequences of the COVID-19 pandemic. People resigned from medical health care services due to the lockdown, and their resignation was related to panic fear of pandemic and irrational behaviors, which delayed diagnosis and treatment [19,20].

Without any doubt, the epidemy of SARS-Cov2 caused an excess number of deaths in Poland from the beginning of 2020. However, despite the role that COVID-19 played in those excessed deaths, as well, it should not be refused that the number of other causes of death was less important. It seems that people were more likely to die because of cardiovascular diseases or respiratory diseases due to resignation from medical services or difficulty access to specialists or general practitioners.

## 5. Conclusions

COVID-19 had an impact on the excess number of deaths in Poland in 2020. However, the simulation shows that participation of COVID-19 in total deaths should not exceed 20% for Poland and 24% for the Silesian voivodship in 2020.

## Figures and Tables

**Table 1:** Impact of COVID-19 on excess mortality in particular studies [4,5,6,7,8].

Author/Paper	Excess Deaths	Country	Time	Estimation Attributed to COVID-19
Woolf SH.: Excess Deaths from COVID-19 and Other Causes, March–July 2020	225,530	USA	March–July	67%
Stokes AC.: Assessing the Impact of the Covid-19 Pandemic on US Mortality: A County-Level Analysis	202,000	USA	February–September	26.3% (95% CI, 20.1% to 32.5%)
Kung S.: Underestimation of Covid-19 mortality during the pandemic	210,959	Europe 14 countries	January–September	62% (range: −254.5% to 197.1%)
	505,657	Rest of the world 8 countries	January–September	52% (range: −4.6 to 78.5%)
	Total			
	716,616	22 countries all over the world	January–September	35–65%
Shiels.: Impact of Population Growth and Aging on Estimates of Excess U.S. Deaths during the COVID-19 Pandemic	218,000	USA	March–August	80%
Krelle H.: Understanding excess mortality What is the fairest way to compare COVID-19 deaths internationally?	38,499	Wales and England	March–April	71%

**Table 2:** Yearly and monthly number of total death registered between 2017 and 2020 in Poland.

Year	Number of Deaths in Poland												
	Total	January	February	March	April	May	June	July	August	September	October	November	December
2017	405,404	43,778	37,352	34,902	32,032	31,972	30,155	30,848	30,997	30,929	34,426	32,463	35,550
2018	414,074	37,822	37,080	41,787	34,639	32,452	30,752	32,741	31,877	31,330	34,274	32,961	36,359
2019	410,981	39,020	36,382	36,117	33,614	33,229	33,079	32,822	31,971	31,721	34,178	32,809	36,039
2020	478,658	36,560	35,250	36,863	34,116	33,703	32,637	33,537	34,773	34,181	49,343	64,474	53,493
%_2017_	18.1↑	16.5↓	5.6↓	5.6↑	6.5↑	5.5↑	8.2↑	8.7↑	12.1↑	10.4↑	43.3↑	98.6↑	50.5↑
%_2018_	15.6↑	3.3↓	5.0↓	11.8↓	1.5↓	3.8↑	6.1↑	2.4↑	9.1↑	9.1↑	43.9↑	95.6↑	47.1↑
%_2019_	16.4↑	6.3↓	3.1↓	2.1↑	1.5↑	1.4↑	1.3↓	2.1↑	8.7↑	7.7↑	44.3↑	96.5↑	48.4↑

Legend: in the last three lines, the % represents the percentage change (decrease or increase) in the number of deaths in 2020. Compared to previous years. ↑: increase; ↓: decrease.

**Table 3:** Yearly and monthly number of total death registered between 2017 and 2020 in Silesian voivodship.

Year	Total Deaths in Silesian Voivodship												
	Total	January	February	March	April	May	June	July	August	September	October	November	December
2017	51,404	5773	4781	4429	4095	4014	3796	3891	3850	3931	4288	4181	4375
2018	52,159	4669	4707	5270	4314	4020	3840	4187	3874	4051	4381	4184	4662
2019	51,766	4990	4668	4470	4116	4204	4186	4177	4036	4006	4260	4135	4518
2020	60,105	4689	4612	4735	4296	4355	4104	4245	4514	4299	6283	7746	6227
%_2017_	17.0↑	19.0↓	3. 5↓	7.0↑	5.0↑	8.5↑	8.1↑	9.0↑	17.2↑	9.4↑	46.5↑	85.2↑	42.3↑
%_2018_	15.2↑	0.4↓	2.0↓	10.0↓	0.4↓	8.3↑	7.0↑	1.4↑	16.5↑	6.1↑	43.4↑	85.1↑	33.6↑
%_2019_	16.1↑	6.0↑	1.2↓	5.9↑	4.4↑	3.6↑	2.0↓	1.6↑	11. 8↑	7. 3↑	47.3↑	87.3↑	37.8↑

Legend: in the last three lines, the % represents the percentage change (decrease or increase) in the number of deaths in 2020. Compared to previous years. ↑: increase; ↓: decrease.

**Table 4:** Total and COVID-19 deaths in Poland in 2020.

Month	A1	A2	A3	A4	A5	A6	A7	A8	A9	A10	A11	A12	A13	A14	A15
	An Average Value in the Period 2017–2019	Deaths in 2020 (*n*)	Reported Deaths COVID-19 (*n*)	% of COVID-19 Deaths in Total Deaths (2020)	Excess Deaths	Proportion of COVID-19 Deaths in Excess Deaths (%)	First Scenario 30% of Excess Deaths (*n*)	First Scenario Reported + Estimated COVID-19 Deaths (*n*)	First Scenario Estimated COVID-19 Deaths in Total Deaths 30%+ Reported Deaths (%)	Second Scenario 60% of Excess Deaths (*n*)	Second Scenario Reported + Estimated COVID-19 Deaths (*n*)	Second Scenario Estimated COVID-19 Deaths in Total Deaths 60%+ Reported Deaths (%)	Third Scenario 70% of Excess Deaths (*n*)	Third Scenario Reported + Estimated COVID-19 Deaths (*n*)	Third Scenario Estimated COVID-19 Deaths in Total Deaths 70%+ Reported Deaths (%)
	A1	A2	A3	A3/A2	A2 − A1	A3/A5	30% × A5	A7 + A3	A8/A2	60% × A5	A10 + A3	A11/A2	70% × A5	A13 + A3	A14/A2
March	37,602	36,863	33	0.1	−739	n/a	n/a	n/a	n/a	n/a	n/a	n/a	n/a	n/a	n/a
April	33,428	34,116	610	1.8	688	88.7	206	816	2.4	412	1023	3.0	482	1091	3.2
May	32,551	33,703	421	1.2	1152	36.5	345	767	2.3	691	1112	3.3	806	1227	3.6
June	31,559	32,637	400	1.2	1078	37.1	323	723	2.2	646	1047	3.2	755	1154	3.5
July	32,186	33,537	252	0.8	1351	18.7	405	657	2.0	810	1063	3.2	946	1197	3.6
August	31,592	34,773	323	0.9	3181	10.2	954	1277	3.7	1908	2232	6.4	2227	2549	7.3
September	31,326	34,181	473	1.4	2855	16.6	856	1330	3.9	1713	2186	6.4	1999	2471	7.2
October	34,292	49,343	3119	6.3	15,051	20.7	4515	7634	15.5	9030	12,150	24.6	10,536	13,654	27.7
November	32,744	64,474	11,519	17.9	31,730	36.3	9519	21,038	32.6	19,038	30,557	47.4	22,211	33,730	52.3
December	35,982	53,493	11,401	21.3	17,511	65.1	5253	16,654	31.1	10,506	21,908	41.0	12,258	23,658	44.2
Total	333,262	407,120	28,551	7.0	73,858	38.7	22,157	50,708	12.5	44,314	72,866	17.9	51,701	80,251	19.7

**Table 5:** Total and COVID-19 deaths in the Silesian voivodship in 2020.

Month	A1	A2	A3	A4	A5	A6	A7	A8	A9	A10	A11	A12	A13	A14	A15
	An Average Value in the Period 2017–2019	Deaths in 2020 (*n*)	Reported Deaths COVID-19 (*n*)	% of COVID-19 Deaths in Total Deaths (2020)	Excess Deaths X	Proportion of COVID-19 Deaths in Excess Deaths (%)	First Scenario 30% of Excess Deaths (*n*)	First Scenario Reported + Estimated COVID-19 Deaths (*n*)	First Scenario Estimated COVID-19 Deaths in Total Deaths 30%+ Reported Deaths (%)	Second Scenario 60% of Excess Deaths (*n*)	Second Scenario Reported + Estimated COVID -19 Deaths (*n*)	Second Scenario Estimated COVID-19 Deaths in Total Deaths 60%+ Reported Deaths (%)	Third Scenario 70% of Excess Deaths (*n*)	Third Scenario Reported + Estimated COVID-19 Deaths (*n*)	Third Scenario Estimated COVID-19 Deaths in Total Deaths 70%+ Reported Deaths (%)
	A1	A2	A3	A3/A2	A2 − A1	A3/A5	30% × A5	A7 + A3	A8/A2	60% × A5	A10 + A3	A11/A2	70% × A5	A13 + A3	A14/A2
March	4723	4735	8	0.16	12	66.6	4	16	0.3	7	19	0.4	8	20	0.4
April	4175	4296	110	2.56	121	90.9	36	157	3.6	72	193	4.4	84	231	5.3
May	4079	4355	90	2.06	276	32.6	83	359	8.2	165	441	10.1	193	366	8.4
June	3940	4104	121	2.94	164	73.7	49	213	5.1	98	262	6.3	114	285	6.9
July	4085	4245	61	1.43	160	38.1	48	208	4.8	96	256	6.0	112	221	5.2
August	3920	4514	88	1.94	594	14.8	178	772	17.1	356	950	21.0	415	682	15.1
September	3996	4299	76	1.76	303	25.0	91	394	9.1	182	485	11.2	212	379	8.8
October	4309	6283	372	5.92	1974	18.8	592	2566	40.8	1184	3158	50.2	1381	2346	37.3
November	4166	7746	1303	16.83	3580	36.3	1074	4654	60.0	2148	5728	73.9	2506	4883	63.0
December	4518	6227	1175	18.86	1709	68.7	513	2222	35.6	1025	2734	43.9	1196	2884	46.3
Total	41,911	50,804	3404	7.01	8893	38.2	2668	6072	11.9	5335	8740	17.2	6225	12,297	24.2

## Data Availability

The data is available free of charge from national reports (Polish Statistical Office).
